# Potential Role of Natural Antioxidant Products in Oncological Diseases

**DOI:** 10.3390/antiox12030704

**Published:** 2023-03-12

**Authors:** Pasquale Marino, Giacomo Pepe, Manuela Giovanna Basilicata, Vincenzo Vestuto, Stefania Marzocco, Giuseppina Autore, Alfredo Procino, Isabel Maria Gomez-Monterrey, Michele Manfra, Pietro Campiglia

**Affiliations:** 1 Department of Science, University of Basilicata, Viale dell’Ateneo Lucano 10, 85100 Potenza, Italy; 2 Department of Pharmacy, University of Salerno, Via G. Paolo II, 84084 Fisciano, Italy; 3 Department of Pharmacy, University of Naples Federico II, Via Pansini, 80131 Naples, Italy

**Keywords:** cancer, chemoprevention, nutraceuticals, carotenoids, polyphenols, antioxidant compounds

## Abstract

Nutrition has a significant effect and a crucial role in disease prevention. Low consumption of fruit and vegetables and a sedentary lifestyle are closely related with the onset and development of many types of cancer. Recently, nutraceuticals have gained much attention in cancer research due to their pleiotropic effects and relatively non-toxic behavior. In fact, although in the past there have been conflicting results on the role of some antioxidant compounds as allies against cancer, numerous recent clinical studies highlight the efficacy of dietary phytochemicals in the prevention and treatment of cancer. However, further investigation is necessary to gain a deeper understanding of the potential anticancer capacities of dietary phytochemicals as well as the mechanisms of their action. Therefore, this review examined the current literature on the key properties of the bioactive components present in the diet, such as carotenoids, polyphenols, and antioxidant compounds, as well as their use in cancer therapy. The review focused on potential chemopreventive properties, evaluating their synergistic effects with anticancer drugs and, consequently, the side effects associated with current cancer treatments.

## 1. Introduction

“Let food be thy medicine and medicine be thy food”.

Since ancient times, it has been known that lifestyle and diet have an important influence on human health and well-being, as well as playing a crucial role in the prevention of disease. Incorrect dietary habits associated with a sedentary lifestyle have proved to be contributory causes of various pathologies, from cardiovascular disease to cancer [1]. The discipline known as “Nutraceutical” studies “the food components or the active ingredients present in foods that have positive effects for well-being and health, including the prevention and treatment of diseases”. This term was coined in 1989 and is derived from the combination of the terms “nutrition” and “pharmaceutical” [2]. It identifies a food or part of a food, which can be of vegetal or animal origin, that has a beneficial pharmaceutical activity beyond its nutritional value. Nutraceutics are often confused with food supplements, which do not have a specific pharmacological effect on health.

Using nutraceuticals in the daily diet prevents the onset of pathological conditions by avoiding or reducing the use of drug therapy; therefore, it is considered “beyond the diet, before the drug” [3].

**Figure 1:**
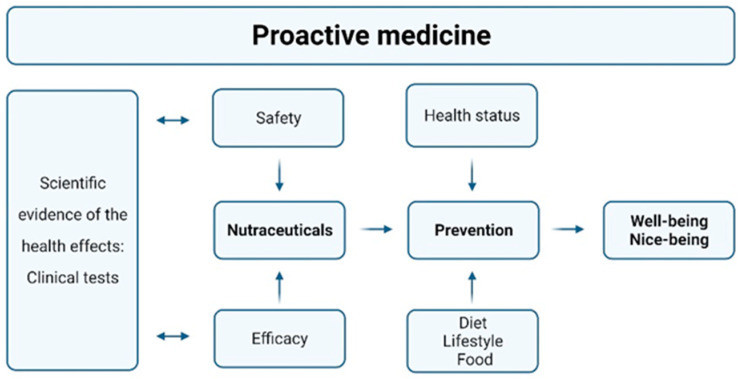
Proactive medicine nutraceutical approach (Created with “https://www.biorender.com (accessed on 12 December 2022)”.

Nowadays, the focus has shifted toward prevention of disease rather than providing a cure or therapy. For these reasons, there is a growing interest in alternative and more “natural” approaches to preventive actions with respect to pharmacologic therapy [4,5]. This is why nutraceuticals can be considered important elements in “proactive medicine”. Approaches that seek to prevent the onset of disease rather than pharmacologically treat the symptoms are becoming increasingly evident. Numerous studies and clinical trials have confirmed the efficacy and safety of the use of nutraceuticals. These studies, coupled with a healthy and balanced lifestyle, demonstrate the preventative role of these substances, which protect against the development of disease and maintain individuals’ well-being. Figure 1 summarizes the overall nutraceutical approach to health and prevention.

**Figure 2:**
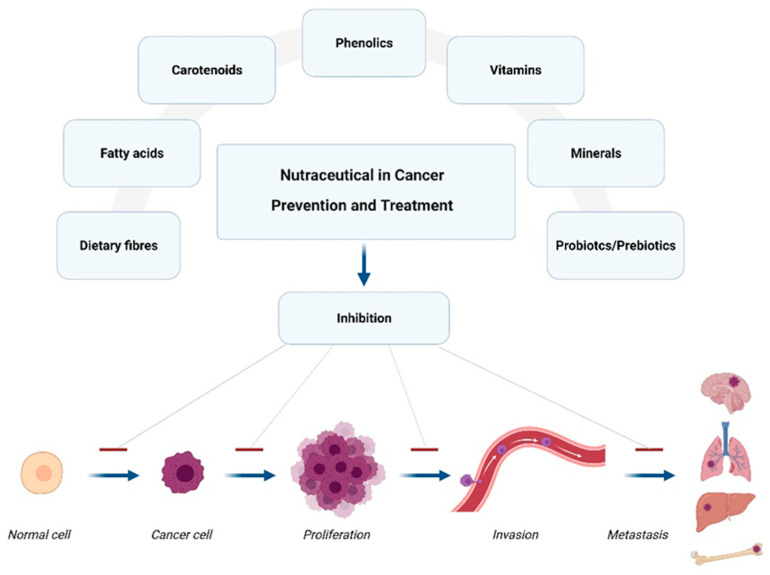
Schematic illustration of various kinds of nutraceuticals used in cancer prevention and treatment (Created with “https://www.biorender.com (accessed on 12 December 2022)”.

Recently, nutraceuticals have gained much attention in cancer research because of their pleiotropic effects and relatively non-toxic behavior [6]. Cancer is caused by a complex, multistep process in which cumulative genetic and epigenetic changes occur in a normal cell. Cancer development is characterized by three stages: initiation, promotion, and progression [7]. The first stage in tumor development is initiation, a rapid and irreversible process during which an alteration of the cell’s genetic material stimulates the cell to become cancerous. Genotoxic DNA damage can be spontaneous or caused by an exogenous or endogenous carcinogen, which involves the activation of oncogenes or the inactivation of tumor suppressor genes. Promotion is a process characterized by the transformation of a preneoplastic cell initiated by agents that stimulate its proliferation, such as growth factors, hormones, and UV radiation. This leads to the transmission of the transformed genotype to daughter cells and the accumulation of further mutations. The final stage of neoplastic transformation, progression, involves uncontrolled cell growth, increased invasiveness and metastatic potential [7]. Between 30 to 40% of cases are reportedly preventable through diet modification, adequate body weight management, and physical activity [8]. The use of nutraceuticals in cancer therapy is mainly aimed at chemoprevention to reduce drug resistance, identifying synergistic effects with anticancer drugs to decrease drug concentrations and, consequently, the side effects associated with current anticancer treatments. Chemopreventive compounds must be safe, inexpensive, and accessible and they must have clear and defined molecular mechanisms. Nutraceuticals that are useful for health include essential compounds, such as selenium, zinc, calcium, and vitamins C, D, E, and B as well as non-essential compounds, such as carotenoids, polyphenols, conjugated acids, and fatty acids. These are obtained from different sources, such as plants, fruits, and animals (Figure 2) [9]. Nutraceuticals act in cancer prevention and treatment by inhibiting cancer cells’ proliferation and differentiation, inhibiting efflux transporters such as P-glycoprotein (P-gp), or reducing chemotherapy drugs’ toxicity, such as cardiotoxicity or hepatotoxicity. They also act on cell cycle control, apoptosis, inflammation, angiogenesis, and metastasis. Furthermore, natural compounds can also have an antioxidant activity by protecting against free radicals, which alter the structure of DNA and cell membranes [10]. The study of this subject is known as onconutraceutics, a specific nutraceutical branch that examines the interactions and actions of the bioactive components of food matrices against cancer.

Many species of plants and fruit in the Mediterranean have shown high onconutraceutical potential thanks to the action of their bioactive compounds. These species include *Malus domestica*, *Vitis vinifera*, *Citrus sinensis*, *Punica granatum*, *Allium sativum* L., *Humulus lupulus* L., *Rosmarinus officinalis* L., *Aloe barbadensis*, *Brassicaceae* [11,12].

The aim of this review is to report the studies on the main phytochemical properties and to evaluate their possible use in cancer chemoprevention in anticancer therapy with an additive or synergistic action (adjuvant therapy). This review also aims to decrease anticancer drug concentrations to limit the adverse effects of anticancer therapies, and to delay resistance to therapy (chemo-, hormone-, and radiotherapy).

## 2. Onconutraceutics: Experimental Studies on the Use of Substances of Natural Origin in Cancer

### 2.1. Carotenoids

**Figure 3:**
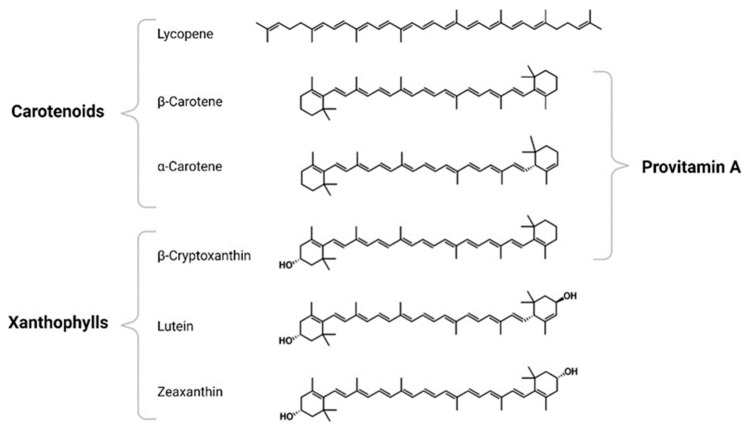
Structure of the reviewed carotenoids.

Many studies have shown that various carotenoids have been associated with a protective effect against many cancers. Carotenoids are a family of compounds of over 600 lipophilic plant pigments, whose structure is based on a C_40_ isoprenoid backbone. This can be linear or modified to have cyclic structures at one or both ends, bearing different functional groups [13]. These lipophilic compounds are responsible for the yellow, orange, and red colors of many fruits and flowers without chlorophyll, and for the coloring of many species, such as plants, fungi, algae, and bacteria. Lycopene, β-carotene, α-carotene, lutein, zeaxanthin, and β-cryptoxanthin (Figure 3) are the most common carotenoids in human serum [14].

Common carotenoid sources are brightly colored fruits and vegetables. The main food sources of dietary lycopene are tomatoes and tomato products, as well as fruits such as watermelon [15,16]. β-carotene is found in orange and yellow fruits, as well as green leafy vegetables such as carrots, mangoes, and kale. Like β-carotene, α-carotene is found in orange and yellow fruits and green leafy vegetables such as pumpkins, carrots, and tomatoes at lower levels [17]. Lutein and zeaxanthin are found in a variety of vegetables and egg yolk, with kale, spinach, and broccoli containing the highest amount. β-cryptoxanthin can be found in a variety of dietary sources such as papaya, pumpkin, egg yolk, butter, and apples (Table 1) [18].

**Figure 4:**
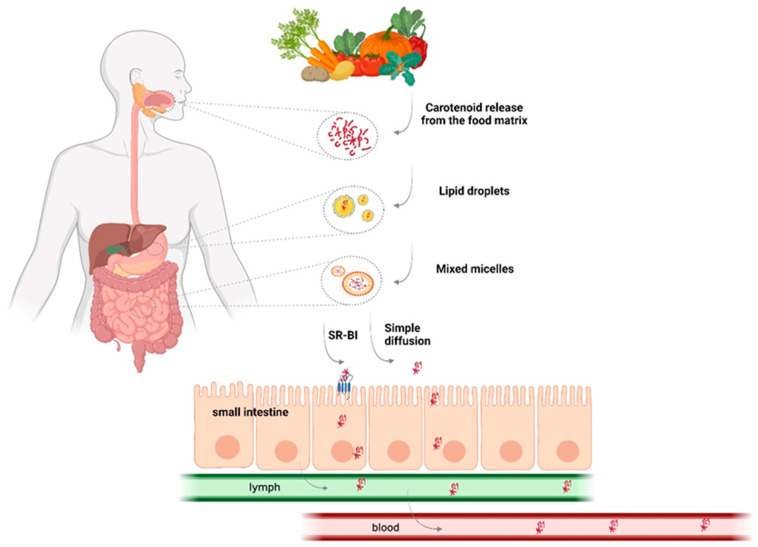
Schematic representation of the absorption of carotenoids (Created with “https://www.biorender.com (accessed on 12 December 2022)”.

Due to their largely hydrocarbon structure, carotenoids tend to be non-polar and need dietary fat to be absorbed into the intestinal lumen [19]. Once released from their food matrix, carotenoids are incorporated into the lipid phase and emulsified in small lipid droplets in the stomach. Then, they are incorporated into micelles formed by bile salts, bile phospholipids, dietary lipids, and the action of their hydrolysis products. The micelles subsequently migrate to the intestinal epithelium where the carotenoids are absorbed by the enterocytes. Intestinal absorption of carotenoids occurs mainly through a passive diffusion mechanism. However, some studies suggest the existence of active transport mediated by scavenger receptor class B type I (SR-BI receptor) [20,21]. Once absorbed, the carotenoids are packaged in chylomicrons, secreted into the lymphatic system, and transported to the liver [14] (Figure 4).

Many studies have evaluated the safety of carotenoid supplements in cancer patients. A group of 36 prostate cancer patients was enrolled in a Phase I-II study and treated with increasing doses of lycopene. The subjects were divided into 6 groups and each group was given a different dose of lycopene (15, 30, 45, 60, 90, and 120 mg/day) for 1 year. Toxicity was mild and only 1 patient discontinued therapy due to severe diarrheal phenomena (grade 2 toxicity). Significant increases in plasma lycopene concentration were observed 3 months after supplementation. This study determined that lycopene supplementation in subjects with prostate cancer was found to be safe and well tolerated [22]. Another study established that the safety level for lycopene consumption was 75 mg per day, while the level for lutein was 20 mg per day [23]. β-carotene is generally recognized as safe and used as a supplement and nutrient [24]. Hence, carotenoid supplementation is well tolerated by most people.

**Figure 5:**
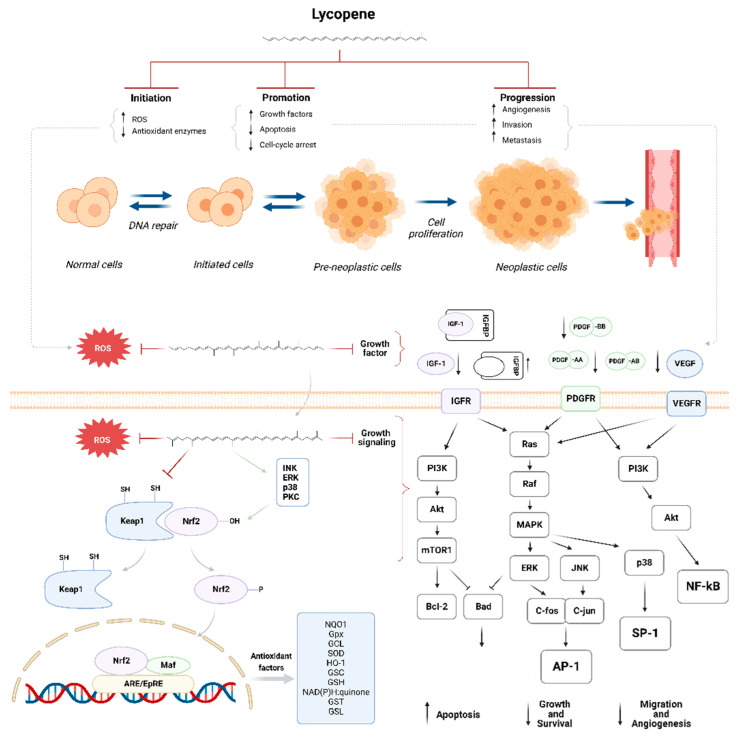
(**Top**) Phases of lycopene intervention in the carcinogenic process. (**Bottom left**) A possible mechanism of Nrf2 regulation by lycopene. (**Bottom right**) Lycopene induces cell-cycle arrest and apoptosis (Created with “https://www.biorender.com (accessed on 12 December 2022)”.

The major mechanisms through which carotenoids have been implicated in cancer are related with pathways involving cell growth and death. Lycopene induces both cell cycle arrest and apoptosis in tumor cells. Growth factors, such as platelet-derived growth factor (PDGF) and insulin-like growth factor (IGF), enhance cell survival by protecting cells from apoptosis. Platelet-derived growth factor receptors (PDGFRs) and insulin-like growth factor receptors (IGF-IRs) are activated at the cell surface during tumorigenesis. The activation of these receptors induces several downstream signaling pathways that regulate the expression of the genes that are important for proliferation, cell-cycle progression, apoptosis, invasion, and metastasis. Lycopene directly binds to PDGF to reduce the autophosphorylation of PDGFR. Furthermore, lycopene has been shown to inhibit the IGF-induced activation of IGFR by increasing the expression of IGF-binding proteins (IGFBPs). Moreover, lycopene blocks cell-cycle progression from G1 to S phase and increases the levels of the tumor suppressor, p53. It also promotes apoptosis by decreasing Bcl-2 and survivin expression, increasing the levels of the proapoptotic proteins Bax, Bad, Bim, and Fas ligand, as well as activating caspases 8, 9, and 3 (Figure 5) [25].

Natural carotenoids display strong antioxidant capacity [26]. Lycopene and its metabolites block the “initiation” phase by neutralizing reactive oxygen species (ROS) by activating detoxification systems and the expression of antioxidant enzymes, thereby protecting cells from damage caused by carcinogenic substances. Lycopene can also block or prevent tumor promotion and progression by modulating inflammatory cytokines and growth factor pathways (Figure 5).

A previous study proposed that the effects of lycopene may be attributed to the induction of antioxidant proteins and phase II detoxifying enzymes [27]. Nuclear factor erythroid 2–related factor 2 (Nrf2) is an important transcription factor that plays a crucial role in the detoxification of carcinogenic agents and antioxidant cellular defense systems.

Under physiological conditions, Nrf2 is sequestered in the cytoplasm by inhibitory protein kelch-like ECH-associated protein 1 (Keap1), a cytoskeletal protein which in turn serves as a substrate for cullin 3 (Cul3), a marker of the ubiquitination system. By continuously labeling Nrf2 with ubiquitin, Cul3 accelerates its 26S proteasome degradation, preventing the overexpression of target genes controlled by the Nrf2/Keap1 system. However, in case of oxidative stress, the electrophilic species release generates the complex formation between sulfhydryl groups of the Keap1 cysteine residues. This causes the detachment of the Keap1 protein from Nrf2, that translocated into the nucleus, resulting in the upregulation of cytoprotective gene sequences and recruiting co-activators of the Maf protein family [25]. In addition, Nrf2 nuclear translocation may also be indirectly activated by bioactive compounds through the phosphorylation of its Ser groups by several kinases (e.g., c-Jun N-terminal kinases (JNKs), mitogenic activated protein kinases (MAPKs), glycogen–synthase kinase-3β(GSK3β), protein kinase C (PKC), protein kinase R (PKR)-like endoplasmic reticulum kinase (PERK)) implicated in transducing redox signals (Figure 5) [28,29].

#### 2.1.1. Carotenoids and Prostate Cancer

Many studies have correlated the antitumor activities of carotenoids in prostate and breast cancer.

Prostate cancer is the most commonly diagnosed cancer in men. The development of prostate cancer is complex, and androgens are an important hormone in the growth and progression of prostate cancer. A study evaluating the results of the lycopene use on genes related with prostate cancer determined that the genes involved in androgen metabolism pathways were significantly downregulated [30].

Lycopene, β-carotene, and α-carotene are the most widely studied carotenoids for prostate cancer. As demonstrated in a meta-analysis that contains data from 42 studies, including 43,851 prostate cancer cases and 692,012 participants, an increase in lycopene consumption and blood concentration reduced prostate cancer risk with a dose-linear response. As lycopene consumption increased for every additional 2 mg dose, the relative risk of prostate cancer decreased by 1% (*p* = 0.026), while for each additional 10 μg/dL^−1^ circulating lycopene, the risk decreased by around 4% (*p* < 0.004) [31].

Another meta-analysis of 34 studies, including 15,891 cases and 592,479 participants, showed that an increase in α-carotene consumption resulted in a 13% lower relative risk of developing prostate cancer and a 2% reduction for each additional 0.2 mg of α-carotene consumed [18,32].

However, no significant associations were found between circulating α-carotene, dietary β-carotene, or circulating β-carotene and prostate cancer risk.

#### 2.1.2. Carotenoids and Breast Cancer

Breast cancer is the most commonly diagnosed cancer in women. In a meta-analysis of studies on six carotenoids [33], it was found that only dietary β-carotene, blood α-carotene, and blood lutein were associated with breast cancer reduction. These findings are extensively described in Aune et al. (2012), and taken up by Rowles III and Erdman Jr. in 2020 [18,33,34]. These studies suggest that β-carotene, α-carotene, and lutein may be associated with improved breast cancer survival.

#### 2.1.3. Carotenoids and Gastric Cancer

Although the incidence of gastric cancer (GC) is reduced, it is still the fifth most common cancer in the world. Several risk factors for GC have been identified, such as *Helicobacter pylori* infection and tobacco smoke [35,36]. Moreover, eating behaviors can affect about 35% of all different tumors, so changing eating habits is a determining factor in the prevention of several cancers, including GC. Therefore, the study and identification of specific nutrients, together with a correct diet, play a crucial role in the prevention of GC [37,38,39]. In 2018, a report published by the World Cancer Research Fund (WCRF) confirmed that the intake of fruit and vegetables could be closely related with the reduction of GC risk [40]. It has been shown that, among the more than 40 carotenoids present in different foods, 6 types of primary carotenoids are isolated from the human blood (α-carotene, β-carotene, β-cryptoxanthin, lutein, zeaxanthin, and lycopene). This confirms the selective intestinal adsorption of these carotenoids [41,42,43]. Some studies show that the intake of carotene through healthy foods is able to reduce the risk of GC. In particular, the intake of β-carotene has been associated with decreased GC, mainly through the suppression of ROS-mediated inflammatory processes, including mitogenic activated protein kinases (MAPKs), redox-sensitive transcription factors, and reduced expression of inflammatory mediators [44,45,46,47]. Further research has demonstrated that increasing the food intake of lycopene could be inversely associated with the risk of GC. In particular, in patients with risk factors such as being *H. pylori*-positive or a history of smoking, the assumption of lycopene showed a positive correlation with a decrease in GC risk [48,49,50].

#### 2.1.4. Carotenoids and Lung Cancer

Lung cancer is the second most diagnosed cancer and is the most common cancer mortality factor among men and women in the United States [51]. The main risk factor for lung cancer onset is cigarette smoking. Smokers often have other risk factors, including poor diet (reduced consumption of fruit and vegetables) [52,53]. Carotenoids can reduce oxidative stress. Therefore, the intake of carotenoids through a healthy diet could exert a protective and/or preventive action against the risk of lung cancer [54,55]. A 2007 report by the WCRF and the American Institute for Cancer Research (AICR) measured blood concentrations of lycopene, α-carotene, β-carotene, β-cryptoxanthin, and lutein to demonstrate a correlation with lung cancer [56]. This report, which was subsequently updated by Abar et al. (2016), revealed that lycopene and carotene intake was related with an approximately 30% reduction in the risk of developing lung cancer. Moreover, using a dose-response assay, it has been shown that by increasing lycopene in the blood to values of 10 μg dL^−1^, the risk of lung cancer can be reduced by 10%. Similarly, by increasing carotene in the blood to values of 20 μg dL^−1^, the risk of lung cancer can be reduced by 16%. Finally, with the same dose–response test conducted with α-carotene, a 34% reduction in lung cancer risk has been shown for each additional dose in the blood equal to 5 μg dL^−1^ [18,56].

#### 2.1.5. Carotenoids and Colorectal Cancer

Colorectal cancer (CRC) is the third most commonly diagnosed cancer and the third leading cause of cancer mortality in men and women [51]. It has been shown that increasing the food intake of fruit and vegetables promotes the assumption of carotenoids; in particular, it has been shown that lycopene exerts a preventive action against the risk of neoplastic transformation of the colorectal tract. HT-29 colon cancer cells treated with lycopene were able to slow down cell proliferation. Further research conducted on animal models has shown that lycopene can control the suppression and progression of colorectal cancer, mainly by inhibiting the phosphatidylinositol 3-kinase (PI3K) and Akt factors [57]. Furthermore, lycopene intake was able to upregulate the expression of proliferating cell nuclear antigen (PCNA) and p21, while at the same time downregulating cyclooxygenase-2 (COX-2), prostaglandin E2 (PGE2), and the phosphorylated protein extracellular signal-regulated protein kinases (ERK1/2) in tumor-bearing mice [58]. These findings suggest that carotenoids, particularly lycopene, may exert a positive effect by blocking the growth and progression of colorectal cancer. Nonetheless, a prospective study of fruit and vegetable consumption and their relationship with colorectal cancer on 1,743,645 person-years and 937 cases of colon cancer showed that their frequent consumption does not appear to confer protection against cancer [59,60]. However, these findings may depend on the smoking status of patients. In fact, other studies on the association between fruit and vegetable intake and CRC risk based on smoking status show that cigarette smoking may affect the association between fruit and vegetable intake and CRC risk among men [61].

#### 2.1.6. Carotenoids and Pancreatic Cancer

Pancreatic cancer is the fourth leading cause of cancer death among men and women, although it is less common than other types of neoplasia [51]. Smoking, alcohol, family history, and type 2 diabetes are crucial factors for pancreatic cancer risk. Furthermore, it has been shown that the inflammatory mechanism plays a key role, both during neoplastic transformation and tumor progression [62]. This is because smoking and diabetes are factors capable of triggering an inflammatory response and oxidative stress, while carotenoids exert an action of controlling the inflammatory process. Therefore, increasing the intake of carotenoids should exert a positive effect on the reduction of pancreatic neoplasia risk [62,63,64,65]. In a 2016 study, it was found that higher dietary intake of β-carotene and β-cryptoxanthin was significantly associated with reduced pancreatic cancer risk. However, no significant association was observed for α-carotene, lutein, and zeaxanthin [66].

### 2.2. Polyphenols

In the multiplicity of phytochemicals, polyphenols represent the largest group with numerous different chemical structures. Polyphenols are compounds with at least one aromatic ring functionalized with one or more hydroxyl groups. Natural polyphenols are a large group of secondary metabolites in plants. They include small molecules and highly polymerized compounds that protect plants from disease, infection, and damage [67,68]. Polyphenols are widely used in foods and drinks of plant origin. Depending on the inter-connection and the number of phenolic rings, polyphenols are collected in different classes (Table 2) [14].

Flavonoids are among the most abundant classes. They are classified into anthocyanins, flavonols, flavones, flavanones, isoflavones, flavanols, and flavonolignans. Phenolic acids are the second most common class of polyphenols and are present in coffee and black tea. They are primarily classified as benzoic and cinnamic acid derivatives. Stilbenes are not always present in plants but generally produced after injury or infection. Finally, lignans are phytoestrogens that are highly abundant in flaxseed and flaxseed oil (Table 3) [69]. An important factor for the bioavailability and efficacy of polyphenolic compounds is their molecular structure. Polyphenols have been shown to exhibit numerous activities. Polyphenols play an important role in preventing chronic disease, from cardiovascular and neurodegenerative diseases, to diabetes and cancer [4]. Furthermore, polyphenols scavenge for free radicals, reduce oxidative stress, and modulate different signaling pathways [70].

**Figure 6:**
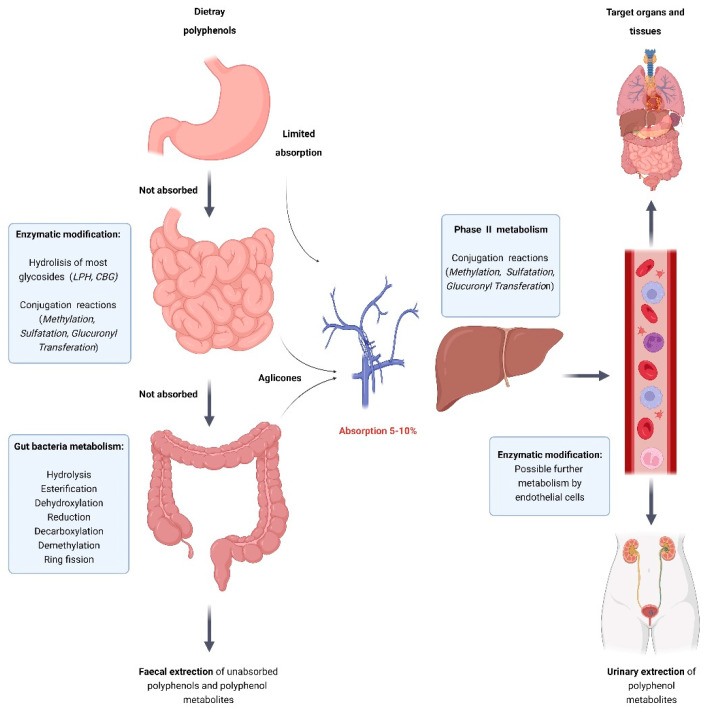
Schematic representation of the absorption and metabolism of food polyphenols (Created with “https://www.biorender.com (accessed on 12 December 2022)”.

A limitation in the use of polyphenolic compounds is due to their bioavailability. Generally, after oral ingestion, polyphenols are present in low concentrations in the blood and urine as they undergo various metabolic changes and steps. They initially undergo a first enzymatic modification and a metabolic process by the intestinal microflora. They are then primarily metabolized in the liver by methylation, glucuronidation, and sulfation. Subsequently, they are rapidly excreted in bile and urine (Figure 6). Therefore, only a small portion reaches the organs and tissues (Table 3) [71].

Several polyphenols have shown high onconutraceutical activity. Epigallocatechin gallate (EGCG), the most abundant polyphenol in green tea extract, has been found to have anticancer activity in various types of cancer. EGCG is the most studied flavanol against CRC. Studies have shown an important action of EGCG in blocking the activation of epidermal growth factor receptor (EGFR), thereby inhibiting tumor growth. EGCG has also been shown to reduce β-catenin levels and increase E-cadherin levels by reducing tumor cell motility [72,73]. Furthermore, EGCG has demonstrated antitumor activity by reducing the levels of COX-2 and proinflammatory cytokines, such as tumor necrosis factor alpha (TNF-α) [74]. Other studies have demonstrated the ability of EGCG to induce cell cycle arrest in the G1 phase and increase apoptosis in various colorectal cancer cell lines [75,76].

Resveratrol is one of the most widely studied polyphenols against CRC. Resveratrol is a polyphenol belonging to the stilbenes class and is also the most studied compound. Resveratrol occurs naturally, mainly in grapes and red wine. It has been identified as the most promising multi-target antitumor agent in red wine, and is important in both cancer prevention and treatment.

Studies have shown that resveratrol displays anti-cancer activity at different carcinogenesis stages, from initiation to metastasis. It also has antioxidant and anti-inflammatory properties that reduce the lesions induced by oxidative stress. It has been shown that by preventing the action of epidermal growth factor (EGF), resveratrol suppresses the initiation, promotion, and progression of carcinogenesis. This action induces the reduction of vascular endothelial growth factor (VEGF) expression, reducing angiogenesis and the formation of more aggressive tumor forms. Inflammation is also a critical component of tumor progression; in this context, by inhibiting inflammatory processes, resveratrol could be used in the treatment of cancer [77]. Indeed, Ren et al. demonstrated that resveratrol suppresses TNF-α-induced signaling in a dose-dependent manner, both via nuclear factor kappa-light-chain-enhancers of activated B cells (NF-κB) activation and via p65 transcriptional activity. In addition, resveratrol appears to play a role in inhibiting tumor invasion and metastases [78].

It has been shown that some flavonoids exert a protective effect against several tumors. The rationale of these studies was based on the ability of flavonoids, which are present in foods, to act as regulators of the inflammatory process and cell proliferation, and to act as negative regulators of the mutagenic process [79]. A recent study was conducted to demonstrate that lung cancer risk was inversely associated with the intake of 5 subclasses of flavonoids (anthocyanidins, flavan-3-ols, flavones, flavonols, and flavanones). However, the generic data obtained were disappointing, and no association was found between flavonoid intake and lung cancer risk reduction. However, reduced intake of flavones and flavanones was associated with an increase in squamous cell carcinoma, but not adenocarcinoma [80]. It has recently been shown that resveratrol is able to regulate metastatic processes. The rationale for this mechanism is based on the ability of resveratrol to control, directly and/or indirectly, epithelial–mesenchymal transition (EMT) by acting on the PI3K/AKT/NF-κB pathway and downregulating the levels of E-cadherin, N-cadherin, vimentin, matrix metalloproteinase-2 (MMP-2), and matrix metalloproteinase-9 (MMP-9), which is fundamental in motility and metastasis processes [81]. In particualr, an in vitro study was conducted on A549 lung cancer cells treated with resveratrol and transforming growth factor-1 (TGF-1) (factor promoting EMT). The data show that the capability of this polyphenol to block the EMT process occurs mainly by means of TGF-1 inhibition; therefore, it has been assumed that resveratrol may block the development of metastases by inhibiting EMT [82]. Although the study results are still contradictory, the results of experimental work have shown a positive relationship between the intake of flavonoids and the prevention of GC. GC is still today the fourth leading cause of cancer mortality in the world. This neoplasia affects twice as many males as females [83]. Although experimental data are still limited, a correlation appears increasingly evident between the intake of flavonoids in the diet and protection of the gastrointestinal mucosa. This is probably caused by the ability of these molecules to exert an antimutagenic and anticarcinogenic action. At present, several case-control studies confirm that the assumption of flavonoids reduces the risk of GC [82,84,85,86,87].

Hepatocarcinoma (HCC) represents the sixth most common cause of cancer-induced mortality in the world [77]. In vitro studies have demonstrated the anticancer effect of flavonoids in HCC cell lines [80,88,89]. Furthermore, the use of animal models has demonstrated the ability of flavonoids to regulate cellular processes such as (i) cell proliferation, (ii) angiogenesis, and (iii) control of tumor metastases [90]. Compared with in vitro studies and animal model studies, there is limited data on humans. In particular, a case-control study has demonstrated decreased HCC risk induced by the assumption of flavonoids [91]. Moreover, a much larger cohort study seems to confirm the association between reduced HCC and the intake of flavonoids.

Some studies have considered the safety evaluation of the use of these natural compounds. Polyphenols have been shown to be harmless and well tolerated, and cases of toxicity associated with their consumption are rare. Most noteworthy are 2 reports of grade 4 toxicity reactions associated with the consumption of green tea polyphenols and quercetin. Toxicity was assessed monthly using the Common Toxicity Criteria (CTC). Grade 4 toxicity was shown to be severe or medically significant, but not immediately life-threatening, with hospitalization or prolonged hospitalization indicated. The first report concerned the daily consumption of 6 g of green tea extract. One patient with advanced prostate cancer presented grade 4 toxicity, manifested as confusion requiring a 5-day hospital stay. The second was seen during a study with increasing doses of quercetin, administered intravenously. One episode of Grade 4 nephrotoxicity occurred at the weekly dose of 1.4 mg/m^2^. Doses of quercetin associated with toxicity were significantly higher than the average dietary intake, estimated to be ~16 mg/day. This toxicity could be explained by the lower absorption of quercetin compared with other polyphenolic compounds, which resulted in elevated circulating levels [92].

Numerous studies have evaluated the efficacy of dietary polyphenols against cancer [93]. It has been shown that polyphenols can interfere in the carcinogenesis process through various mechanisms, such as blocking the bioactivation of carcinogenic compounds. They can also induce the apoptosis of cancer cells [94].

Several studies have also been carried out on the effects and risks of high dietary polyphenol intake on cancer. Evidence from epidemiological studies (Table 4) has already been well documented and discussed in the work of Zhou et al. (2016). Analyzing various cancer types and cell lines, numerous epidemiological studies have yielded conflicting results, as shown in Table 4. In fact, many studies have demonstrated a significant association of polyphenols with reduced cancer risk, while others have shown an insignificant association, particularly data obtained from studies on breast, colorectal, and gastric cancer [95]. Several factors could explain the controversial results of in vivo studies of the efficacy of plant-derived compounds. Unlike a pharmaceutical composed of a single and concentrated active principle used for single therapeutic activity, plant extracts are exceedingly complex, multicomponent mixtures composed of many molecules with different chemical–physical properties. Moreover, the data comparison of in vivo pharmacological effects of natural substances obtained from different clinical trials is complicated by chemical composition variability of natural extracts. In fact, the qualitative and qualitative profiles of bioactive compounds depend both on intrinsic factors (e.g., plant genus and species) and extrinsic factors (e.g., cultivation practices, production methods, growing conditions, environmental and climatic factors, stability during processing, and storage).

The health benefits of plant-based extracts are often related to the synergistic action of their constituents that may act on one or more molecular targets. Therefore, it is necessary to identify their mechanism of action to determine their pharmacokinetic properties and to standardize constituent concentration. This will ensure constant levels of active principles in the plant extract and, consequently, the reproducibility of their pharmacological effects.

**Table 3:** Bioavailability in human of some natural polyphenols (Adapted from [95]).

Polyphenol	Structure	Food	Concentration in Food	Concentration in Plasma
Anthocyanin	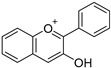	Blackberries	960 µM 200 g^−1^	NA
Naringenin	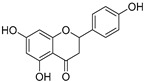	Oranges	7.9 mg 100 g^−1^ FW	Mean Cmax 80 nM, Tmax 5.88 h
Hesperetin	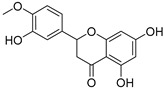	Oranges	53.1 mg 100 g^−1^ FW	Mean Cmax 90 nM, Tmax 7 h
Quercetin	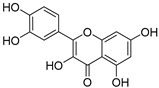	Dry shallot skin	4.9 µmol g^−1^ FW	Mean Cmax 3.95 µM, Tmax 2.78 h
Daidzein	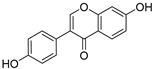	Soy milk	2.2 mg 100 mL^−1^	196.1 nM after 5 days
Genistein	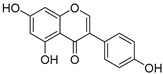	Soy milk	6.8 mg 100 mL^−1^	797.04 nM after 5 days
Ellagic acid	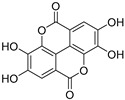	Blackberries Raspberries	300 µg g^−1^ DW	Mean Cmax 10 nM, Tmax 1.98 h
Gallic acid	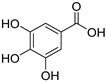	Grape skin	700 µg g^−1^ DW	NA

FW: fresh weight. DW: dry weight. NA: not available.

**Table 4:** Epidemiological Studies: dietary polyphenol intake and cancer risk (Adapted from [95]).

Polyphenols	Cancer	Risk Estimates (95% CI)
Flavonoids	Breast cancer	flavonols 0.88 (0.80–0.98) # flavones 0.83 (0.76–0.91) # no significant association for total flavonoids or other subclasses #
	Colorectal cancer	no significant association ^§^
		0.59 (0.35–0.99) *
	Gastric cancer	0.33 (0.15–0.73) *
		no significant association *
	Lung cancer	0.63 (0.47–0.85) *
	Prostate cancer	total catechin 0.73 (0.57–0.95) ^§^ epicatechin 0.74 (0.57–0.95) ^§^ kaempferol 0.78 (0.61–1.00) ^§^ myricetin 0.71 (0.55–0.91) ^§^
Flavanols	Breast cancer	0.81 (0.67–0.97) ^§^
	Hepatocellular carcinoma	0.62 (0.33–0.99) ^§^
Isoflavones	Breast cancer	0.68 (0.52–0.89) #
	Colorectal cancer	0.76 (0.59–0.98) #

case-control study *. cohort study ^§^. meta-analysis #.

#### 2.2.1. Polyphenols and Prostate Cancer

Many studies point to the efficacy of green tea catechins (GTCs) in the prevention of prostate cancer. A study by Bettuzzi et al. on high-grade prostate intraepithelial neoplasia volunteers demonstrated the safety and efficacy of catechins in the chemoprevention of prostate cancer. In this double-blind, placebo-controlled study, 30 men were treated with 600 mg/day of GTC (epigallocatechin (EGC), 5.5%; epicatechin (EC), 12.24%; EGCG, 51.88%; epicatechin gallate (ECG), 6.12%; total GTC, 75.7%; caffeine, <1%) and 30 with a placebo. After 1 year, only 1 tumor was diagnosed among the 30 GTC-treated men, while 9 tumors were found among 30 placebo-treated men. This study demonstrated the efficacy of green tea catechins in the treatment of premalignant lesions before the development of prostate cancer [96]. A dose–response meta-analysis by Guo et al. [97] on green tea consumption and prostate cancer incidence revealed that each cup/day of increased green tea intake reduced the risk of prostate cancer with a relative risk (RR) of 0.954 (95% confidence interval (CI): 0.903, 1.009) for all studies, 0.989 (95% CI: 0.957, 1.023) for cohort studies and 0.893 (95% CI: 0.796, 1.002) for case-control studies. These studies have shown that consuming more than 7 cups of green tea a day significantly reduces the risk of prostate cancer. The results of a case-control study showed a significant relationship between a high intake of total polyphenols and subclasses of polyphenols in the diet and the risk of prostate cancer. The study showed that treatment with total polyphenol (odds ratio (OR): 0.12; 95% CI: 0.03–0.41), lignans (OR: 0.14; 95% CI: 0.04–0.41), phenolic acids (OR: 0.18; 95% CI: 0.05–0.57), and the intake of certain subgroups of flavonoids, including flavan-3-ols (OR: 0.24; 95% CI: 0.08–0.67), flavanones (OR: 0.10; 95% CI: 0.03–0.31), and flavones (OR: 0.33; 95% CI: 0.12–0.87), significantly reduced prostate cancer risk (*p* < 0.05) [98]. Russo et al. (2017) investigated the association between dietary phenolic acid consumption and prostate cancer, and found that higher intake of hydroxybenzoic and caffeic acids was associated with a lower risk of advanced prostate cancer [99]. A meta-analysis by Guo et al. (2016) showed a significant relationship between high intake of anthocyanidins and reduced prostate cancer risk [100]. Lewis et al. (2009), in a case-control study in Japan, found a significant inverse relationship between high consumption of isoflavones (daidzein and genistein) and the risk of prostate cancer [101], while in another case study control, individuals who received high amounts of genistein and daidzein were 14% and 20% less likely to develop prostate cancer, respectively [102].

#### 2.2.2. Polyphenols and Gastric Cancer

Several reviews have summarized the epidemiological evidence on the association between dietary polyphenol intake and cancer risk, suggesting a reduction in gastric cancer risk following flavonoid intake [103,104]. These benefits of flavonoid consumption could be related to lower risk of GC through various mechanisms, such as antioxidant effect, the blocking of cancer pathways, the induction of apoptosis, and the inhibition of *H. pylori* growth [105]. In fact, a case-control study showed that a high consumption of fruit- and vegetable-derived flavonoids induced a protective effect against the risk of GC [106].

A meta-analysis of prospective studies, including 143 clinical studies by Grosso, Godos et al. (2017), showed that isoflavone intake was significantly associated with the reduced risk of GC [107]. However, the results are often conflicting. Epidemiological studies have found that flavonoid intake has rarely been associated with a reduction in cancer risk, particularly in Asian countries [108,109]. For these reasons, further studies are needed to better understand the association between cancer risk and polyphenol intake.

#### 2.2.3. Polyphenols and Lung Cancer

The intake of fruit and vegetables is thought to protect against lung cancer. Epidemiological and experimental evidence suggests that polyphenols may act as chemopreventive agents that delay, suppress, or reverse cancer progression to advanced lung cancer through various mechanisms. These include antioxidant/anti-inflammatory activities, modulation of biotransformation enzymes, antiproliferative effect, and immune system modulation [110,111]. A meta-analysis of eight prospective and seven case-control studies showed that isoflavone intake was significantly associated with decreased risk of lung cancer (RR 0.91, 95% CI: 0.84, 0.87) [107]. A case-control study in Canada reported favorable effects of high dietary intake of total flavonoids on lung cancer risk [80]. A meta-analysis of five case-control and cohort studies identified an inverse association between dietary quercetin and lung cancer (OR 0.66, 95% CI: 0.47–0.92). The same meta-analysis reported an inverse association with kaempferol and no association with total dietary flavonoids (OR 0.78, 95% CI: 0.64–0.95) [112].

#### 2.2.4. Polyphenols and Colorectal Cancer

In a pooled analysis of the Health Professionals Follow-Up Study (HPFS), the Nurses’ Health Study (NHS I), and the European Prospective Investigation into Cancer and Nutrition (EPIC) study, an inverse association was reported between dietary phenolic acids and colon cancer for men (hazard ratios_log2_ = 0.91, 95% CI 0.85–0.97) but not for women (HR_log2_ = 1.10, 95% CI 1.02–1.19). The same analysis found little evidence of association between other classes of polyphenols and colon cancer [91,113].

Furthermore, a study performed by Mori et al. (2022) showed that the inverse associations of plasma concentrations of 3,4-dihydroxy-phenylpropionic acid, ferulic acid, and caffeic acid (all derived from coffee) was inversely associated with the risk of colon cancer. These results support the possible role of coffee and coffee polyphenols in the prevention of colon cancer development [114]. Furthermore, a case-control study suggested that dietary intake of total flavonoids (OR, 0.59; 95% CI, 0.35–0.99) and lignans (OR, 0.59; 95% CI, 0.34–0.99) could reduce colorectal cancer risk [115]. However, the evidence is also contradictory, with the Fukuoka study reporting no association between total dietary polyphenol and colorectal cancer risk [116].

#### 2.2.5. Polyphenols and Breast Cancer

Numerous studies have highlighted the association between the consumption of polyphenols and the reduced risk of breast cancer. Indeed, according to a meta-analysis, the risk of breast cancer was reduced in women with a high intake of flavonols and flavones [117]. Studies have also suggested that soy isoflavone intake reduces the risk of breast cancer for Asian women, and is greater for postmenopausal women than for premenopausal women. However, this significant association was not found in Western women due to low isoflavone consumption in the Western population [118]. Furthermore, a prospective US cohort study showed that there was a modest inverse trend for dietary flavanol intake and the risk of estrogen receptor negative (ER-negative) but not estrogen receptor positive (ER-positive) breast cancer [119].

One study found that higher intake of lignans was associated with the reduced risk of breast cancer [120]. However, the results of a meta-analysis showed that there was no association between plant lignan intake and overall breast cancer risk. When the analysis focused on menopausal status, a significant reduction was observed in postmenopausal women. The reduction in breast cancer risk observed in postmenopausal women in the study is likely due to the phytoestrogens in lignans replacing some of the missing estrogen and counteracting its decline, which may reduce the risk of developing breast cancer [121]. A meta-analysis of studies reporting outcomes associated with green tea consumption and breast cancer risk revealed a significant protective association between green tea consumption and breast cancer risk. Studies were conducted on a total of 14,058 breast cancer patients and 15,043 control subjects. Individuals who received green tea were found to have a negative association with the risk of future breast cancer (OR 0.83; 95% CI: 0.72–0.96). However, significant heterogeneity was seen among these studies [122].

## 3. Onconutraceutics: Associations with Conventional Chemotherapy

### 3.1. Multidrug Approach

Many studies have demonstrated the relationship between natural origin compounds and conventional chemotherapy. Numerous studies suggest that the most promising approaches to cancer therapy are achieved by a multitarget approach, capable of exploiting different mechanisms of action to attack cancer cells. This action can be achieved through a multidrug regimen, and also by including polyphenolic compounds.

Numerous human studies have been performed to evaluate the potential combination of polyphenols with standard treatments in advanced metastatic colorectal cancer (mCRC). The CUFOX (FOLFOX plus curcumin) study combined daily oral curcumin with FOLFOX-based chemotherapy (5-fluorouracil, folinic acid, and oxaliplatin) in subjects with histologically diagnosed mCRC [123]. The daily addition of oral curcumin to standard chemotherapy was evaluated and found to be safe and tolerable, allowing the use of curcumin in the phase IIa open-label randomized controlled trial. The study highlighted that an increase in median progression-free survival (PFS) and overall survival (OS) was achieved in patients treated with CUFOX compared with the control group [124].

Recently, the first human study of genistein in combination with FOLFOX therapy or FOLFOX/bevacizumab was approved for the initial treatment of mCRC [125]. Subjects initially received six cycles of therapy in combination with oral genistein, and subsequently received six additional cycles or underwent surgical resection. The response rate and PFS obtained in this pilot study were substantially better than those previously reported for chemotherapy alone [126], suggesting that combined treatment may improve efficacy.

A prospective proof of concept study evaluated the administration of regorafenib in combination with a complex of silybin, Vitamin E, and phospholipids in patients with mCRC. The study revealed that silybin increases the clinical efficacy and tolerability of regorafenib, leading to an increase in PFS and OS; a median PFS of 10.0 months and a median OS of 17.6 months were observed in these patients [127]. The data suggest that the use of silybin combined with regorafenib could be a promising strategy to improve the efficacy of this drug, whose toxicity has limited its use in clinical practice.

Cao et al. (2019) reported on the efficacy of the *Astragalus* and chemotherapy combination in advanced non-small cell lung cancer (NSCLC) by integrating data from 19 clinical trials involving 1635 participants. *Astragalus* is a traditional Chinese medicinal herb capable of stimulating the immune system, and is considered generally safe. The meta-analysis data indicate a positive effect of the combination with cisplatin. This is because the objective response rate was 19% higher in the group receiving the extract combined with chemotherapy compared with the group receiving chemotherapy alone (RR = 1.19, 95% CI [1.06, 1.33], *p* = 0.002. In addition, *Astragalus* could reduce the cisplatin side effects, such as leukopenia (RR = 0.52, 95% CI: 0.44, 0.61; *p* < 0.00001) and vomiting (RR = 0.72, 95% CI: 0.60, 0.87; *p* = 0.0006), thereby improving compliance with chemotherapy and the patient’s quality of life [128].

A meta-analysis conducted by Lin et al. (2019) reported data on the efficacy of *Astragalus*-based drugs and chemotherapy combination in colorectal cancer. The data from this meta-analysis covered 22 studies and 1409 patients. The results showed a positive effect of this combination, which could increase the efficacy of chemotherapy and improve the patient’s quality of life by reducing many side effects, including neutropenia, anemia, thrombocytopenia, nausea, vomiting, diarrhea, and neurotoxicity. However, there are several limitations to this meta-analysis: first, the provenance of the *Astragalus* preparations was not described in the meta-analysis. Second, many clinical trials to which the data relate are not registered; they should have been registered in a clinical trial registry prior to enrolling subjects. Therefore, the results are not very reproducible [129].

### 3.2. Antioxidative Dietary Compounds

There is significant interest in the safety of antioxidant food supplements in cancer patients and their possible impact on anticancer therapies. Despite their beneficial effects (e.g., side effect reduction and adjuvants in anticancer treatment), antioxidant agents could decrease the effectiveness of treatments by reducing their induction of oxidative stress [130]. However, there are contradictory results on the ability of these compounds to act as allies against cancer. Two studies, one from *Science Translational Medicine* and another from *Nature*, seem to indicate that antioxidant compounds may be harmful. In the first study [131], which was conducted on a xenograft melanoma mouse model, N-acetylcysteine (1 g/L) was administered to drinking water in a standard dose in mouse studies, corresponding to 114–229 mg/kg body weight for an adult male mouse. This corresponded to 665–1330 mg/day in a human weighing 70 kg. The study found that N-acetylcysteine was able to accelerate the formation of metastases. In the second study [132], Morrison and colleagues provided additional evidence, showing that antioxidants can promote cancer metastasis. In mouse models of melanoma, treatment with antioxidants decreased oxidative stress in the circulating cancer cells, thereby increasing their ability to metastasize. Over the past twenty years, some trials published in the *New England Journal of Medicine* and the *Journal of the National Cancer Institute* have also been suspended: lung cancer patients who were administered β-carotene had a higher mortality rate than those in the control group [133,134].

However, many other studies have shown the effectiveness of antioxidant compounds against cancer. Selenium supplements are frequently used by cancer patients. Selenium is an essential trace element and, given its similarity to sulfur, it is involved in antioxidant protection and redox-regulation in humans. These antioxidant effects can mainly be attributed to redox active selenoproteins acting as ROS detoxifying and chelators agents [135].

Several adverse effects of radiotherapy and chemotherapy in cancer patients have been linked to oxidative cell processes in the human body. It has been claimed that selenium alleviates the side effects of conventional cancer therapy, and that, indeed, it protects against cisplatin-induced nephropathy and neuropathy. Furthermore, it has been observed that the severity of the undesirable effects is reduced when selenium is included in treatment as an additional dosage [136].

Vitamin E has been shown to inhibit multiple cancer-promoting pathways, including COX and 5-lipoxygenase (5-LOX); transcription factors, such as NF-κB and signal transducer; as well as activators of transcription factor 3 (STAT3). Vitamin E has an antiproliferative action on cancer cells through the modulation of various signaling pathways, including the metabolism of sphingolipids [134,137]. Vitamin E inhibits tyrosine kinase from inducing apoptosis, in addition to improving chemotherapy results by increasing transforming growth factor-β (TGF-β) release and inhibiting growth signals in malignant cells. Studies have shown a significant reduction of toxicity for doses of 300 to 600 mg of Vitamin E in several types of cancer. The interest in Vitamin E as a preventive factor against prostate cancer began with the Alpha-Tocopherol, Beta-Carotene Cancer Prevention (ATBC) study, which showed a 35% risk reduction in smokers treated with tocopherol and carotene [133,138]. In fact, a data analysis of the Carotene and Retinol Efficacy Trial (CARET) study showed lower α-tocopherol levels in patients with prostate cancer than in control subjects who did not develop the disease [139].

The Selenium and Vitamin E Cancer Prevention Trial (SELECT) study by Klein et al. on the use of selenium and Vitamin E in prostate cancer prevention was stopped prematurely after about 5 years; 473 cases of cancer were highlighted in the group treated with Vitamin E, 416 in the controls with placebo, 432 with selenium alone, and 437 with selenium and Vitamin E. The same analysis revealed an increased risk of type 2 diabetes mellitus in the selenium group. After the interruption of treatments in 2008, 521 new cases of prostate cancer were recorded in the monitoring until 2011 [138]. These data demonstrate conflicting results in the use of these compounds.

Vitamin C, a potent water-soluble antioxidant, is among the most common dietary supplements in cancer patients. Epidemiological studies have highlighted conflicting results on the role of Vitamin C in cancer [140] and in cancer therapy. Several preclinical studies suggest that Vitamin C may increase the efficacy of anticancer agents [141,142]. Vitamin C cancer action could also be associated with its oxidation byproducts, resulting from the oxidized form dehydroascorbic acid (DHA). These induce a mechanism dependent on hydrogen peroxide formation with ascorbate radical as an intermediate [143,144]. The selective toxicity could be ascribed to cancer tissues overexpressing glucose transporters (GLUTs), the same transporters used by DHA to enter cancer cells [145].

Furthermore, the results of a meta-analysis conducted by Zang et al. (2020) suggest that a high intake of total Vitamin C appears to be significantly correlated with a reduced risk of breast cancer incidence, mortality and recurrence [146].

In a 2012 Cochrane review of randomized controlled trials using supplements of Vitamin A, Vitamin C, Vitamin E and selenium, there was no reduced risk in the general population but a small and significant increase in this risk with β-carotene administered to smokers and subjects exposed to asbestos [147]. In addition, 34 of 46 studies reviewed in 2016 suggest that the use of antioxidant vitamins and minerals administered after cancer diagnosis reduce chemotoxicity and radiotoxicity [148]. The question remains whether the use of antioxidants can reduce the effectiveness of radiotherapy or therapies with cisplatin that generate cytotoxic radicals.

In contrast to these data, numerous studies have also demonstrated many possible anti-cancer benefits of Vitamin D, in particular, [149] as well as fish oils [150,151]. This vitamin is biosynthesized from 7-dehydrocholesterol, which, absorbs ultraviolet B radiation to produce pre-Vitamin D_3_ during sun exposure. Once formed, this thermodynamically unstable product rearranges to form Vitamin D_3_ (cholecalciferol). The first hydroxylation reaction takes place in the liver, with the formation of 25-hydroxyVitamin D; in the kidney, it becomes 1,25-dihydroxyVitamin D, which is the active form (calcitriol) in the endocrine, autocrine, and paracrine sense. In fact, Vitamin D acts as a hormone by modulating inflammation and immune system activity. In particular, inhibition of angiogenesis represents one of the main mechanisms used by Vitamin D in its anti-cancer properties since it reduces the expression of angiogenetic factors, such as VEGF, hypoxia-inducible factor 1 (HIF-1), and interleukin-8 [152]. Sun exposure provides about 500 IU/min of Vitamin D, while eggs and fish contain about 200–400 IU/day. Several studies have demonstrated its anti-cancer activity through the induction of apoptosis. A study showed that 30 nmol/L is minimum level, 50 nmol/L is sufficient, while 100 nmol/L can become toxic. It is therefore recommended to take a maximum of 50 µg/day. These studies have shown that patients with such Vitamin D levels have a better prognosis for many cancers (breast, lymphoma, stomach, colorectal, lung, prostate, melanoma, and leukemia) [153].

The Marshall study (2012) reported data on 4000 IU/day Vitamin D supplementation in patients with low-grade prostate cancer. Of the 44 patients who completed the study and received Vitamin D, 24 experienced tumor shrinkage (55%), 5 experienced no change (11%), and 15 patients showed an increase in tumor size (34%). Of the 19 patients who did not receive Vitamin D but placebo, 4 patients experienced tumor shrinkage (21%), 3 experienced no change (16%), and 12 patients experienced tumor enlargement (63%) [154].

A study by McCullough et al. examined 5706 colorectal cancer patients and 7107 control patients with wide levels of circulating 25-hydroxyVitamin D (25(OH)D) concentrations. The results showed that higher blood levels of 25(OH)D were associated with a statistically significant reduction in women’s colorectal cancer but lower risk that was not statistically significant in men. The optimal 25(OH)D concentration to obtain colorectal cancer risk reduction was between 75–100 nmol/L, which is higher than recommended [153].

The randomized phase 2 trial, SUNSHINE, investigated oral supplementation with Vitamin D_3_ added to standard chemotherapy in 139 patients. These patients had unresectable advanced or metastatic colorectal cancer, not previously treated, and all were receiving standard treatment with the mFOLFOX6 (folinic acid, leucovorin, 5-fluorouracil, and oxaliplatin) chemotherapy regimen and bevacizumab. Patients treated with high-dose Vitamin D received an oral dose of 8000 IU/day of Vitamin D_3_ for 2 weeks followed by 4000 IU/day, while patients in the low-dose group received a Vitamin D_3_ standard dose of 400 IU/day. The Phase II trial showed that high-dose Vitamin D supplementation significantly improved progression-free survival (PFS) of colorectal cancer by approximately 2 months compared with a lower dose (13 vs. 11 months). The median follow-up was 16.9 months in the high-dose group and 17.9 months in the low-dose group. Patients in the high-dose group were less likely to experience progression or death at any time during follow-up. The disease control rate in the high-dose group was 96% vs. 84% in the low-dose group (*p* = 0.05). The high-dose did not increase toxicity and the incidence of diarrhea (grade 3 and 4) was even significantly lower in the high-dose group (12% vs. 1%; *p* = 0.02) [155].

In subjects with stage I to III luminal gastrointestinal carcinoma, with more sites affected by metastases or with KRAS wild type tumors, Vitamin D_3_ appears to have a significant protective effect in patients with a lower body mass index. Another factor that highlights a statistically significant improvement in survival is the patient’s age. In fact, considering the age group, a supplementation of 2000 IU/day of Vitamin D_3_ has positive effects (hazard ratio (HR) relapse-free survival, 0.66). These results were highlighted in the AMATERASU study conducted in Japan on 417 patients [156,157].

Vitamin D must be taken in combination with magnesium, because together, they are essential in reducing tumorigenesis and cardiovascular risk. Vitamin D should also be combined with vitamin K2 (100 µg/day) to avoid any extra bone calcifications [158,159,160].

Furthermore, observational studies have shown that magnesium intake affects the status and metabolism of Vitamin D. A clinical study carried out by Dai et al. showed that magnesium supplementation in adults increases the 25-hydroxyVitamin D_3_ (25(OH)D_3_) concentration, but not 24,25-dihydroxyVitamin D_3_ (24,25(OH)_2_D_3_) when baseline 25(OH)D levels are close to 30 ng/mL. Instead, magnesium supplementation decreases the concentration of both in a dose-dependent manner when the 25(OH)D baseline level is higher (30 to 50 ng/mL). Magnesium treatment significantly affected the 24,25(OH)_2_D_3_ concentration when the baseline 25(OH)D concentration was 50 ng/mL, but not 30 ng/mL. These results suggest that maintaining optimal magnesium concentration is necessary to optimize Vitamin D levels [161].

The existing evidence supports the hypothesis that the gut microbiota plays a crucial role in immunotherapy and reduces the radiotherapy and chemotherapy side effects. Studies suggest that gut microbes play a significant role in cancer therapy by modulating drug efficacy, reducing the toxicity and adverse effects of cancer therapy, and mediating toxicity. Moreover, probiotics significantly reduce dysbiosis so as to rebalance the gastrointestinal tract structure and functions, which is the basis of the immune response [162].

The bioactive molecules of some plants can exert their action not only “epigenetically” but also favoring the qualitative and quantitative composition of the intestinal microbiota. For this reason, the World Health Organization (WHO) recommends “a diet based on plants and fermented foods” capable of positively modifying the qualitative and quantitative microbiota composition, which favors a good immune response. The microbiota has an important influence on the immunotherapy effectiveness, another cancer treatment type that is now standard for different disease types. It has been observed that patients in whom immunotherapy is more effective have a much richer intestinal microbiome than other species, while patients who are resistant to immunotherapy have a more limited microbiome. A study directed and coordinated by Humanitas, published in *Nature Microbiology* (2020), demonstrates for the first time that an intestinal bacteria strain (*Erysipelotrichaceae*) plays a protective role against colorectal cancer development; in fact, the absence of this bacteria strain was observed in the preclinical model of colon adenoma. If present, this strain would protect against cancer [163]. Moreover, an increase in cancer “marker” bacteria such as *Fusobacterium nucleatum* was associated with colon cancer [164].

The colon microbiota produces acetic, lactic, butyric, and propionic acid. In particular, butyrate, produced by *Butyricicoccus* and *Clostridium*, is the main energy source of colonocytes and plays a key role in the well-being of the colon [165]. Butyrate increases mitochondrial respiration and ATP consumption. This is a histone deacetylase inhibitor, which is important in tumor progression. It is considered a protective agent capable of blocking cell proliferation and inducing apoptosis, increasing miR-106b-25, miR-18b-106a and miR-17–92 expression, as demonstrated in colon HCT116 cells treated with butyrate [166].

Probiotics are live non-pathogenic microorganisms which, taken in adequate quantities, confer considerable benefits to the host’s health by rebalancing the qualitative–quantitative composition by restoring the intestinal microbiota. Probiotics exert their beneficial effects through various mechanisms, including lowering intestinal pH, decreasing pathogenic organisms colonization and invasion, and modifying the host’s immune response [167].

Intestinal microbiota reconstitution is essential for the good functionality of the immune system. It must be present in about 90% of the intestine to ensure a good immunotherapy response and anticancer chemotherapy. The first controlled and randomized clinical trials on the therapeutic usefulness of probiotics have been underway since 2018. In particular, they are concerned with how probiotics can modulate the intestinal microbiota and immune function in lung cancer patients who need chemotherapy, as well as in breast cancer and pediatric cancer patients [168,169].

## 4. Onconutraceutics: Reduction of Side Effects Associated with Chemotherapy and Radiotherapy 

Although the treatment of malignant tumors with cytotoxic radiotherapy or chemotherapy is effective, it has important short- and long-term side effects, including nephrotoxicity, neurotoxicity, cardiotoxicity, hematological toxicity, gastrointestinal toxicity, and hepatotoxicity [170]. To effectively reduce the side effects induced by chemotherapy and improve cancer patients’ quality of life, excellent results have been obtained from natural products [171].

### 4.1. Oral Mucositis

Clinically significant side effects include oral mucositis (OM). Through a complex biological process involving oral epithelium lesions, oral bacterial super infections and pro-inflammatory cytokines alteration such as TNF-α, iterleukin-1 (IL-1), interleukin-6 (IL-6), OM can manifest as severe ulceration and fungal infection of the mouth (e.g., oral candidiasis). Along with nausea and vomiting, OM induces delay in cancer treatment [172,173].

The WHO reports five levels of severity: grade 0, no change; grade 1, pain/erythema; grade 2, erythema, ulcers, can eat solids; grade 3, ulcers, requires only a liquid diet; grade 4, feeding not possible [174]. Currently, there is no specific protocol to prevent or treat OM; however, basic oral hygiene protocols, 0.12% chlorhexidine digluconate, anti-inflammatory therapy, biological response modifiers, cryotherapy, low-intensity laser therapy, and the use of plant extracts can relieve symptoms [175,176]. The research focuses in particular on phytochemicals.

Many natural products have shown some potential onconutraceutical properties and a possible association with anticancer therapy for an additive or synergistic action, as well as decreasing the concentration of anticancer drugs and the adverse effects of cancer therapies.

In the mucositis guidelines provided by the Mucositis Study Group of the Multinational Association of Supportive Care in Cancer and International Society of Oral Oncology (MASCC/ISOO), the use of cryotherapy for 30 min is recommended during chemotherapy to reduce the absorption of 5-fluorouracil in mucosal cells [177]. This is determined by the vasoconstriction of vessels in the oral cavity caused by cryotherapy [178]. In fact, scientific studies (Cochrane review) report the oral cryotherapy efficacy in reducing oral mucositis of any severity level in individuals’ receiving 5-fluorouracil therapy for solid tumors [179]. It should be noted that oral mucositis affects 40% of patients who use cryotherapy in therapy with standard doses of chemotherapy [180].

*Matricaria recutita Linnaeus* (*Asteraceae*) (*M. recutita*), known as chamomile, is a plant widely used in traditional medicine for its antioxidant, antimicrobial, and anti-inflammatory action. There are no common guidelines on its use; however, if used in the form of antiseptic ointment and mouthwash for 15 days, twice a day, on average, it relieves painful symptoms [181]. Gomes et al. found that all studies considered until April 2018 showed a significant reduction of oral mucositis after using *M. recutita*. It has been shown that *M. recutita* can directly inhibit COX-2 and the synthesis of inflammatory mediators, such as PGE2 [182], thus making it a treatment option for oral mucositis [180].

These results are confirmed by Dos Reis et al. (2016) in a study carried out on 38 patients treated with 5-fluorouracil and leucovorin for gastric or colorectal cancers. The study shows that the anti-inflammatory properties of chamomile flowers can increase the effects of cryotherapy and reduce the onset of oral mucositis [174]. Two other previous clinical studies tested chamomile extract in oral mucositis and neither showed toxicity related to chamomile [183,184]. The strengths of using plant extracts are low cost, no side effects, and easy application [185]. Clinical trials on the effectiveness of *M. recutita* are summarized in Table 5, classified according to the Jadad scale. This is a procedure to independently assess the methodological quality of a clinical trial. It is a system for allocating such trials a score of between zero (very poor) and five (rigorous).

Another interesting natural product for the prophylaxis and treatment of radiotherapy-induced oral mucositis is honey. Its composition basically varies depending on the floral source, but seasonal, environmental factors and processing conditions are also considerable. Honey is composed mostly of the sugars, glucose and fructose, as well as vitamins, minerals, hydrogen peroxide, amino acids, enzymes, organic acids, pollen, fragrance, and flavor compounds [186].

In a systematic review, Münstedt et al. examined 17 randomized trial studies highlighting honey’s ability to attenuate oral mucositis. Of these, 14 claim that honey reduces pain and improves quality of life. However, 4 studies do not associate any positive effect with honey [187]. It should be noted that the honey in question is manuka honey. Manuka honey contains very high amounts of methylglyoxal, a cytotoxic substance that can cause increased DNA modification, contributing to cell and tissue dysfunction, resulting in aging and disease [188,189]. In summary, these studies demonstrate the efficacy of conventional honey, but not manuka honey, in reducing oral mucositis symptoms and radiotherapy and chemotherapy side effects [190].

Currently, microorganisms are found to play important roles in OM and many other diseases, such as diarrhea induced by radiotherapy. A systematic review of the Cochrane Library demonstrated that probiotics might benefit patients with diarrhea resulting from pelvic radiotherapy [191]. Probiotics are live microorganisms that benefit populations and treat specific diseases by improving microbial flora [192,193]. Previous studies have also shown that probiotics exert various activities, from regulating epithelial and mucosal barrier function to protecting against pathogenic bacteria [194]. Probiotics could reduce the incidence and severity of cancer therapy-induced OM, especially for patients with head and neck cancer (HNC). A study by Shu et al. (2020) summarizes and discusses the possible mechanism of action of probiotics [195]. This hypothesis was also confirmed by Ciorba et al. (2012), who showed that the protective effects of *Lactobacillus rhamnosus* GG (LGG), a probiotic, on epithelial cells were lost in myeloid differentiation primary response 88 (Myd88) -/-, toll-like receptor 2 (TLR2) -/-, and COX-2 -/- in mice [196].

Based on current studies, it is still unclear whether it is safe or not for cancer patients to receive probiotics for OM treatment. Many factors need to be considered, including probiotic strain type, dosage, time of use, and patient selection.

Numerous studies have demonstrated the therapeutic effect of *Aloe vera* (AV) in the treatment of oral lesions. AV is a plant belonging to the *Liliaceae* family. Parenchymal cells produce a colorless, mucilaginous gel that consists of 98–99% water and 1–2% active compounds (Table 6). This gel has various properties, such as anti-inflammatory, antioxidant, and anticancer activities. For these reasons, it is traditionally used and has no adverse effects [197].

A study conducted by Bhalang et al. on acemannan extracted from *Aloe vera* demonstrated a reduction in oral aphthous ulcers after 7-day treatment. However, the results were lower than the standard treatment with 0.1% triamcinolone acetonide (TA) [198]. These results are supported by Babaee et al., who demonstrated the efficacy of 2% *Aloe vera* oral gel in the treatment of patients with minor recurrent aphthous stomatitis (RAS) [199]. Ahmadi also claims that aloe vera mouthwash prevents the development of radiation-induced mucositis [200]. Other studies have confirmed the aloe vera treatment efficacy in the therapy against oral mucositis [201] and xerostomia [202,203]. However, the results of Su et al. study differ, with the authors not considering it useful to use *Aloe vera* as an adjuvant to head and neck radiotherapy since it has not shown effects on mucositis or improved patients’ well-being [204]. Despite these conflicting results, *Aloe vera* can be considered effective in the treatment of oral lesions.

### 4.2. Gastrointestinal Toxicity

Chemotherapy-induced vomiting/nausea (CINV) is a painful event for cancer patients, affecting quality of life [205]. The standard treatment to relieve CINV from carboplatin and paclitaxel used in ovarian cancer chemotherapy involves the use of the neurokinin1 receptor antagonists (NK1) combined with the corticosteroids and 5-hydroxytryptamine type 3 (5-HT_3_) receptor antagonists [206]. However, these drugs have an excessive cost. The use of herbal medicine can be a good alternative to reduce the standard treatment use and relieve nausea and vomiting symptoms in malignant gynecological cancer patients. Previous literature revealed that ginger (*Z. officinale*) can reduce nausea and vomiting in pregnancy, postoperative patients, and CINV patients [207,208]. Thanks to the rhizome’s bioactive components, including gingerols, shogaols, zingiberene, zingerone, and paradol, ginger favors intestinal motility normalization. It also modulates the 5-HT3 and NK1 receptors related to the nausea reflex and the emesis derived from the chemotherapy treatment [209]. However, some studies have also revealed adverse reactions, such as dermatitis and indigestion [210]. The Uthaipaisanwong et al. study on gynecological cancer patients receiving a combined carboplatin–paclitaxel regimen demonstrated that adding ginger to chemotherapy brought benefits, especially on the first day, by reducing nausea. The results also show that ginger alone may not be sufficient to control symptoms in the delayed phase [211]. These outcomes are supported by other studies [212,213]. Bossi et al. [214] and Zick et al. [215] showed different results due to the different chemotherapy regimens used.

An integrative review of the literature conducted by Borges et al. reported that low doses of ginger use had greater benefits than higher doses. It also highlighted ginger’s antiemetic efficacy in therapy with the drugs, ondansetron, dexamethasone, and metoclopramide as standard treatment for patients with solid tumors, as well as in combination with palonosetron and dexamethasone [216]. Therefore, the studies show that this complementary therapy is inexpensive, easy to access, and had evident results in nausea and vomiting chemotherapy-induced control.

### 4.3. Nephrotoxicity

One of the main factors limiting chemotherapy in cancer patients is therapy-induced nephrotoxicity. This condition often results in chemotherapy delay or interruption. Nephrotoxicity manifests itself with acute renal injury and hypomagnesaemia, which are associated with defects in filtration, absorption, and excretion. Recently, some studies have evaluated the protective effects of natural products. A study carried out by Osama et al. (2017) examined the use of honey and royal jelly to limit the nephrotoxic effects induced by chemotherapies with platinum-based drugs. The results of this clinical study revealed that cancer patients taking honey and royal jelly capsules had less kidney damage than the control group, as well as lower serum levels of cytotoxic metabolites [217,218]. Consequently, it can be stated that the use of these natural products can help cancer patients during chemotherapy and radiotherapy by improving their quality of life.

### 4.4. Cardiotoxicity

Among the radiotherapy side effects in cancer patients and exposure to ionizing radiation through diagnostic imaging there are radiation-induced heart disease (RIHD). This condition can manifest itself with cardiomyocytes damage that increases the heart attack risk in treated patients. Because of these effects, reduced doses are used, limiting the efficacy of radiotherapy [219,220]. The main damage caused by ionizing radiation are direct DNA structure modification and indirect modification induced by ROS [221,222].

This damage leads to the increased incidence of RIHD in cancer patients. The need to neutralize these effects has led to the development of radioprotective agents. An ideal radioprotector should have minimal toxicity and protect normal tissues, but not cancer cells [223].

A review by Zhang et al. highlighted several natural products that have shown protective effects against chemotherapy-induced cardiotoxicity [224]. The choice of natural products is due to their low toxicity, availability, and affordability. Another review also exhaustively discussed the action of natural products on RIHD [225].

One of the natural products shown to reduce radiation cardiotoxicity is hesperidin. Hesperidin is a flavonoid mainly present in citrus fruits. The effect of this flavonoid was demonstrated by Rezaeyan et al., who showed that the mice treated with hesperidin before exposure to radiation showed a reduction in toxicity conditions and survival improvement [226]. These results confirm the data published by Pradeep et al. (2012) [227].

The radioprotective effect of curcumin and selenium on heart tissue was studied by Kolivand et al. and Amini et al. The results of these studies showed that the combination of natural products before and after exposure to radiation led to a significant reduction in RIHD, modulating the redox system and chronic oxidative stress [228].

Other in vivo studies on mice have shown that resveratrol and quercetin, present in grape juice, prevent RIHD. This is due to their ability to regulate and reduce oxidative damage to the heart (Table 7) [229].

Cardiotoxicity is also a feared side effect that may limit the clinical use of several drugs, such as anthracyclines. This side effect is due mainly to the generation of free radicals from drugs through mitochondrial redox cycling in the cardiomyocytes, which ultimately results in left ventricular dysfunction, and in the most severe cases, congestive heart failure. Several studies showed the cardioprotective efficacy of numerous natural antioxidant products whose beneficial activity has been demonstrated in various models in vitro and in vivo.

Curcumin effects against doxorubicin-induced cardiotoxicity were extensively investigated using rat cardiomyocytes and myocardial injury models in mice. Curcumin treatment reduces lactate dehydrogenase (LDH) and creatine kinase (CK) in serum, as well as suppressing oxidative stress, caspase-3 activity, apoptosis rate, and histopathological changes of the myocardium [230,231].

Catechins are known antioxidants, free radical scavengers, and metal chelators. In vivo, these compounds exhibit positive effects on the myocardium, helping to maintain the appropriate cardiomyocytic architecture and preventing atherosclerosis onset and cardiac hypertrophy [232,233].

*Spirulina platensis*, Reishi (*Ganoderma lucidum*), and *Moringa oleifera* are three nutraceuticals with anti-inflammatory effects that are currently used in cancer patients as complementary and alternative medicines to improve quality of life. In the preclinical models of DOXO-induced cardiotoxicity, these nutraceuticals and their combinations are able to improve cardiac function and reduce biomarkers involved in heart failure and fibrosis [234,235].

## 5. Conclusions

Between 30 to 40% of cancer cases are preventable through diet modification, adequate body weight management, and physical activity. In recent years, the use of nutraceutical compounds as chemopreventive agents is becoming an increasingly interesting approach, as these compounds represent a low-cost, easily accessible, and broad-spectrum alternative to conventional drugs. The aim of this review was to evaluate, by reviewing the literature of the last 20 years, how the knowledge on these compounds has evolved. This review also evaluated their possible use in cancer chemoprevention, in anticancer therapy with additive or synergistic action (adjuvant therapy) to determine a reduction in anticancer drug concentration and the adverse effects of therapies. The literature review considered clinical, in vitro, and in vivo studies. Several nutrient and non-nutrient phytochemicals are being evaluated in intervention studies for their potential as cancer chemopreventive agents.

The use of carotenoid supplements in cancer patients led to a reduction in the risk of developing cancer. In particular, it was found that an increase in the consumption and blood concentrations of lycopene and α-carotene reduced the risk of prostate cancer. Furthermore, dietary β-carotene, blood α-carotene, and blood lutein were found to be associated with a reduction in breast cancer in a meta-analysis of studies. The intake of carotenoids through healthy foods was able to reduce the risk of gastric and colorectal cancer. In particular, the intake of β-carotene and lycopene has shown a positive correlation with a decreased risk of gastric cancer, and can exert a positive effect by blocking the growth and progression of colorectal cancer. Important effects have also been found on lung cancer. The intake of lycopene and carotene was correlated with a 30% risk reduction. Of interest was the dose–response test conducted with α-carotene, which demonstrated a 34% reduction in lung cancer risk for each additional blood dose of 5 μg/dL^−1^. Moreover, the increase in carotenoid intake induced a positive effect in reducing pancreatic neoplasia risk. In a 2016 study, it was found that higher dietary intake of β-carotene and β-cryptoxanthin was significantly associated with reduced pancreatic cancer risk.

Moreover, many studies have suggested a significant relationship between the high intake of polyphenols and subclasses of polyphenols in the diet and the risk of cancer. A meta-analysis of data and case-control studies has demonstrated the efficacy of green tea consumption and green tea catechins in the treatment of premalignant lesions before the development of prostate cancer. Moreover, a case-control study found that individuals who received high amounts of isoflavones, such as genistein and daidzein, were 14% and 20% less likely to develop prostate cancer, respectively. Furthermore, a meta-analysis showed that isoflavone intake was significantly associated with the reduced risk of lung and stomach cancer and, to a lesser extent, breast and colorectal cancer. A meta-analysis of case-control studies showed that flavonoid intake was associated with the reduced risk of gastrointestinal, breast, and lung cancers. However, more studies are needed to better understand the associations between cancer risk and polyphenol intake.

A rather interesting approach to cancer treatment is the use of a multidrug regimen, also including polyphenolic compounds, in combination with typical therapeutic agents. This approach can reduce the aggressiveness and side effects of these drugs, while also improving their effects. The literature has highlighted that the combination of curcumin with FOLFOX in patients with mCRC led to an increase in median progression-free survival and overall survival. Furthermore, the combination of genistein with FOLFOX or FOLFOX/bevacizumab therapy also resulted in better survival and response to therapy, suggesting that combined treatment may improve efficacy. Further evidence is the ability of astragalus to reduce the side effects of cisplatin by improving compliance with chemotherapy and the quality of life of patients with advanced non-small cell lung cancer.

However, there are conflicting results on the ability of some antioxidant compounds to act as allies against cancer. Some studies seem to indicate that antioxidant compounds can promote cancer metastasis. Over the past two decades, some studies have been suspended because lung cancer patients given β-carotene had a higher mortality than those in the control group. However, many other studies have shown the effectiveness of antioxidant compounds on cancer. Studies have shown that patients with optimal Vitamin D levels, around 50 nmol/L, have better prognosis in many cancers (breast, lymphoma, stomach, colorectal, lung, prostate, melanoma, and leukemia). High-dose Vitamin D appears to be particularly useful in colorectal cancer, in combination with magnesium and Vitamin K2. Moreover, probiotics significantly reduce dysbiosis, which could help rebalance the structure and functions of the gastrointestinal tract, underlying the immune response.

An interesting aspect that has emerged about natural products is their ability to effectively reduce the side effects induced by chemotherapy and improve cancer patients’ quality of life. The treatment of malignant tumors with cytotoxic radiotherapy or chemotherapy, while effective, has important short- and long-term side effects, including nephrotoxicity, neurotoxicity, cardiotoxicity, hematological toxicity, gastrointestinal toxicity, and hepatotoxicity. Studies have shown that natural compounds, such as *Matricaria recutita*, honey, probiotics, and *Aloe vera,* can be considered effective in the treatment of oral lesions induced by anticancer therapy and, in particular, oral mucositis, aphthous stomatitis, and xerostomia. Ginger, on the other hand, has been effective in nausea and vomiting chemotherapy-induced controls and is able to improve intestinal mucositis. The results of a clinical study revealed that cancer patients on platinum-based drug chemotherapy who took honey and royal jelly capsules had less kidney damage than the control group, as well as lower serum levels of cytotoxic metabolites. Natural products that have been shown to reduce radiation cardiotoxicity include hesperidin, curcumin, selenium, resveratrol, and quercetin. These results show that natural polyphenolic compounds have remarkable cardioprotective properties against toxicity induced by chemotherapeutic agents. However, the small number of clinical studies and the contradictory results of some studies suggest the need for further studies to better understand the potential anticancer capacities and the mechanisms of action of dietary phytochemicals.

## Figures and Tables

**Table 1:** Carotenoids content per portion (mg) (Adapted from [18]).

Food	Pz (g)	Lyc (mg)	Food	Pz (g)	β-Car (mg)	Food	Pz (g)	α-Car (mg)	Food	Pz (g)	β-Cry (mg)	Food	Pz (g)	Lut-Zea (mg)
Tomato juice, 100%	248	22.4	Carrot juice	240	22.3	Carrot juice	240	10.4	Persimmon	168	2.4	Cooked Spinach	190	29.7
Spaghetti sauce	130	16.5	Baked sweet potato	150	17.2	Cooked pumpkin	245	6.6	Papaya	304	1.8	Cooked Kale	130	25.4
Watermelon	286	13.0	Cooked spinach	190	13.7	Carrots	110	3.8	Mandarin oranges, canned	189	1.5	Dandelion greens	55	7.5
Canned stewed tomatoes	255	10.4	Cooked kale	130	11.4	Cooked winter squash	245	1.7	Red peppers	119	0.6	Chard	36	4.0
Tomatoes	123	3.2	Cooked mustard greens	140	10.3	Plantain	179	0.8	Tangerine	88	0.4	Spinach	25	3.0
Grapefruit	256	2.9	Carrots	110	9.1	Pumpkin bread	60	0.7	Dried Papaya	23	0.2	Kale	25	2.0
Tomato catsup	15	1.8	Parsley	60	3.0	Mandarin oranges, canned	189	0.4	Calamondin	19	0.1	Broccoli	88	1.2
Dried papaya	23	0.7	Kale	25	1.5	Dandelion greens	55	0.2	Kumquat	19	0.04	Cooked egg yolk	17	0.2

Portion size (Pz); Lycopene (Lyc); β-carotene (β-Car); α-carotene (α-Car); β-cryptoxanthin (β-Cry); Lutein (Lut); Zeaxanthin (Zea).

**Table 2:** Polyphenol and secondary metabolite classification and dietary sources.

Classification		Structure	Major Dietary Sources
Flavonoids	Anthocyanins	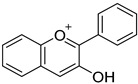	Berries, grapes, cherries, plums, currants, pomegranates, red cabbage
	Flavanols	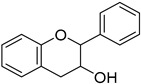	Apples, pears, legumes, tea, cocoa, wine
	Flavanones	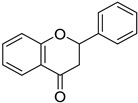	Citrus fruits
	Flavones	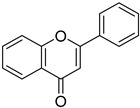	Parsley, celery, orange, onions, tea, honey, spices, oregano
	Flavonols	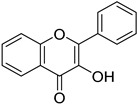	Berries, apples, broccoli, beans, tea, asparagus, leafy greens, onions
	Isoflavones	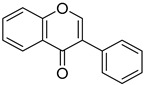	Soy
Phenolic acids	Hydroxybenzoic acids	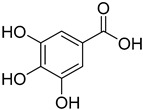	Pomegranates, grapes, berries, guava, blackcurrants, walnuts, chocolate, wine, green tea
	Hydroxycinnamic acids	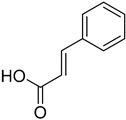	Coffee, cereal grains, tea leaves, red onions
	Lignans	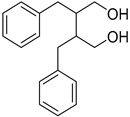	Flaxseed, sesame, barley, buckwheat, oats
	Stilbenes	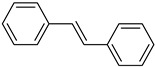	Grapes, berries, red wine

**Table 5:** Clinical trials on the effectiveness of *Matricaria recutita* (L.) for the treatment of oral mucositis (Adapted from [176]).

Jadad Scale	Intervention	Reduction
-	Infusion of dried flowers	Oral mucositis on the 30th day
0	15 drops of chamomile solution diluted in a glass of water	incidence gravidity
3	Gel with 3% of chamomile extract	discomfort severity treatment time
	3 mL of chamomile mouthwash added to half a cup of water	pain incidence
4	Ice with 400 mL of water and 10 g of chamomile flowers	incidence symptomatology ulcerations
	30 mL of chamomile	incidence gravidity
	Kamillan liquidum solution	oral mucositis in 85% of patients
5	Oral antiseptic with 1% of chamomile extract	incidence intensity of injuries oral mucositis duration
	Chamomile Orobase	pain lesion
	Oral antiseptic with 1% of chamomile extract and 1% of peppermint oil	pain dryness of the oral cavity dysphagia

**Table 6:** Major constituents of *Aloe vera* (Adapted from [197]).

Anthraquinones	Inorganic Compounds	Vitamins	Essential Amino Acids	Nonessential Amino Acids	Miscellaneous
Aloin	Calcium	B1	Lysine	Histidine	Cholesterol
Barbaloin	Sodium	B2	Threonine	Arginine	Triglycerides
Isobarbaloin	Chlorine	B6	Valine	Hydroxyproline	Steroids
Anthranol	Manganese	Choline	Leucine	Aspartic acid	β-sitosterol
Aloetic acid	Zinc	Folic acid	Isoleucine	Glutamic acid	Lignins
Ester of cinnamic acid	Chromium	C	Phenylalanine	Proline	Uric acid
Aloe-emodin	Potassium sorbate	α-tocopherol	Methionine	Glycine	Gibberellin
Emodin	Copper	β-carotene		Alanine	Lectin-like substance
Chrysophanic acid	Magnesium			Tyrosine	Salicylic acid
Resistannol	Iron				Arachidonic acid

**Table 7:** Nutraceuticals and their mechanisms of protection against RIHD (Adapted from [225]).

Nutraceutical	Mechanisms of Protection against Radiation-Induced Heart Diseases		
	*Antioxidant*	*Anti-Inflammatory*	*Antiapoptotic*
Hesperidin	√	√	
Curcumin		√	
Melatonin	√	√	√
Selenium	√		
Caffeic acid phenethyl ester	√	√	
Black grape juice	√		
Zingerone	√	√	
